# Microbial community and diversity in the feces of Sichuan takin (*Budorcas taxicolor tibetana*) as revealed by Illumina Miseq sequencing and quantitative real-time PCR

**DOI:** 10.1186/s13568-018-0599-y

**Published:** 2018-04-27

**Authors:** Hui Zhu, Dong Zeng, Ning Wang, Li-li Niu, Yi Zhou, Yan Zeng, Xue-qin Ni

**Affiliations:** 1 Animal Microecology Institute, College of Veterinary, Sichuan Agricultural University, Chengdu, 611130 China; 2 Key Laboratory of Animal Disease and Human Health of Sichuan Province, Chengdu, 611130 China; 3 College of Bioengineering, Sichuan University of Science and Engineering, Yibin, 643000 China; 4 Department of Parasitology, College of Veterinary, Sichuan Agricultural University, Chengdu, Sichuan China; 5 Chengdu Wildlife Institute, Chengdu Zoo, Chengdu, 610081 China

**Keywords:** *Budorcas taxicolor tibetana*, Fecal microbiota, Illumina Miseq sequencing, Quantitative real-time PCR

## Abstract

The Sichuan takin (*Budorcas taxicolor tibetana*) is a rare and endangered ruminant distributed in the eastern Himalayas. However, little information is available regarding the intestinal microbiota of the takin. In this study, Illumina Miseq platform targeting the V4 region of the 16S rRNA was employed to characterize microbial community and diversity in the feces of wild (n = 6) and captive takins (n = 6). The takin exhibited an intestinal microbiota dominated by three phyla: *Firmicutes* (57.4%), *Bacteroidetes* (24.2%) and *Proteobacteria* (12.3%). At family/genus level, *Ruminococcaceae*, *Bacteroidaceae*, *Acinetobacter*, *Clostridium*, *Lachnospiraceae*, *Rikenellaceae*, *Bacillus*, *Comamonas* and *Spirochaetaceae* were dominant. Distinctive microbiotas between wild and captive takins were observed based on microbial community structure, captive takins having significantly higher community diversity. Quantitative real-time PCR were also utilized to monitor predominant bacteria in three Sichuan takin individuals housed in Chengdu Zoo over a half-year period, which showed that microbial communities of the three takins were relatively similar to each other and stable during our study period. Our results suggested that diet was a major driver for shaping microbial community composition.

**Electronic supplementary material:**

The online version of this article (10.1186/s13568-018-0599-y) contains supplementary material, which is available to authorized users.

## Introduction

The microbial populations that reside in the digestive tract of animals are diverse and numerous. Generally, bacteria comprise 40–45% of fecal material on a dry weight basis with populations often exceeding 10^11^ colony-forming unit (CFU) per gram feces (Eckburg et al. 2005; Stephe and Cummings 1980). The bacteria of the animal gastrointestinal tract constitutes a complex ecosystem which is involved in host physiology, ranging from the structure and functions of the digestive system and the innate and adaptive immune systems, to host energy metabolism (Macfarlane and Macfarlane 2004). Conversely, the composition of the intestinal microbiota is also influenced by diet, social interactions, antibiotic use, host anatomy and phylogeny (Russell and Rychlik 2001; Ley et al. 2008b). Although there is a profound relationship between intestinal bacteria and animal health, this ecosystem of many herbivores remains incompletely characterized and its diversity poorly defined.

The takin (*Budorcas taxicolor*), also called cattle chamois or gnu goat, is a rare and endangered species distributed in the eastern Himalayas, which is considered a flag species for wildlife conservation. There are four subspecies of the takin, namely the Mishmi takin (*B. taxicolor taxicolor*), the golden takin (*B. taxicolor bedfordi*), the Sichuan takin (*B. taxicolor tibetana*), and the Bhutan takin (*B. taxicolor whitei*). The Sichuan takin and golden takin are endemic subspecies of China. The body size of the takin is similar to the gaur (*Bos gaurus*) and the wild yak (*Bos grunniens*), but their external characteristics of digestive system are rather similar to sheep according to anatomical records in Chengdu Zoo. As a large ruminant, the takin has a specialized digestive system with a four chambered stomach and a relatively long lower tract (small and large intestine), which are adapted to forage rich in plant cell wall. In systematic zoology, the takin belongs to *Bovidae Ovibovinae* with its close relative, the muskoxen (*Ovibos moschatus*). However, recent mitochondrial researches suggested that takins were more closely related to various sheep and that similarities between takins and muskoxen might be attributed to convergent evolution (Groves and Shields 1997).

Wild Sichuan takins generally are found on forested slopes at 1500–4000 m in elevation. They feed on many kinds of alpine deciduous plants and evergreens, which varies with season and local food availability. This mainly includes the leaves and shoots of bamboo, tough leaves of evergreen rhododendrons and oaks, willow and pine bark, and a variety of new-growth leaves and herbs (Wu and Hu 2001).

The aim of the present study was to characterize the intestinal microbiota of the takin and investigate how diet and environmental factors affect the intestinal microbiota. Since intestinal microbiota have not yet been studied precisely in takin feces to our knowledge, our assays will contribute to enhanced knowledge of the takin intestinal bacteria, for further use in updating the current knowledge of the putative role of intestinal bacteria in health and disease.

## Materials and methods

### Sampling

Fresh feces were collected from 6 wild takins in Labahe Nature Reserve (30°18′N 102°27′E) and 6 captive takins in Chengdu Zoo (Chengdu, Sichuan, China) from late September to October. An adult female takin (subject A, 13-year-old), an adult male takin (subject B, 8-year-old), and a young male takin (subject C, 3-year-old) housed separately at the Chengdu Zoo were also monitored over a half-year period. In January 5, March 2, April 27 and June 19, fecal samples were collected from the three takins respectively. The Sichuan takins housed in Chengdu Zoo were fed a constant and balanced diet, which refer to standards for maintenance requirement of cattle and sheep. Their daily diet of captive animals consisted of 20–25 kg green fodder (perennial ryegrass or Sudangrass), 2.5 kg alfalfa bale, 2 kg chopped alfalfa hay and 2 kg concentrate. The composition of the concentrate was 39% corn, 13% rice bran, 2% fish meal, 4% wheat middling, 15% wheat bran, 8% soybean meal, 12% cracked soybean, 1.5% hydrolyzed feather meal, 1.5% calcium carbonate, 2% dicalcium phosphate, 0.8% sodium chloride and 1.2% microelements and vitamins. For the reason of animal conservation, sampling work was accomplished by specific zoo keepers in Chengdu or staff at the reserve. Fresh fecal samples were immediately collected upon defecation around feeding time or in the process of feeding salts by park rangers. Samples were sealed in sterile plastic bags, transported to the laboratory in liquid nitrogen and then stored at − 70 °C until analysis.

### Ethics statement

All animals were handled in strict accordance with the animal protection law of the People’s Republic of China (a draft animal protection law was released on September 18, 2009). All procedures were performed in accordance with the rules of the Care and Use of Laboratory Animals of the Animal Ethics Committee of Sichuan Agricultural University (Ya’an, China) (approval no. 2013-036). All the methods were carried out in accordance with relevant guidelines and regulations, including any relevant details.

### DNA extraction and sequencing

All samples subjected to DNA extraction were obtained from inside the feces using sterile tweezers to avoid soil contamination with an equal weight of 75 mg. Total genomic DNA was extracted from fecal samples using the E.Z.N.A^®^ Stool DNA Kit (Omega Biotechnology, USA) according to manufacturer’s instruction, and was stored at − 70 °C before further analysis. DNA was amplified by using the 515f/806r primer set (515f: 5′-GTG CCAGCMGCCGCGGTA A-3′, 806r: 5′-XXX XXXGGACTACHV GGGTWT CTA AT-3′), which targets the V4 region of the microbial 16S rRNA, with the reverse primer containing a 6-bp error-correcting barcode unique to each sample. PCR reaction was performed using phusion high-fidelity PCR Mastermix (New England Biolabs LTD., Beijing, China) with the following condition: 94 °C for 3 min (1 cycle), 94 °C for 45 s/50 °C for 60 s/72 °C for 90 s (35 cycles), and a last step of 72 °C for 10 min. PCR products were purified by using the QIAquick Gel Extraction Kit (QIAGEN, Dusseldorf, Germany). Sequencing was conducted on an Illumina MiSeq 2 × 250 platform according to protocols described by Kozich et al. (2013). Sequencing of partial 16S RNA genes was performed by the Novogene Bioinformatics Technology Co., Ltd. (Beijing, China).

### Bioinformatics and statistical analysis

Sample reads were assembled by using Mothur software package (Schloss et al. 2009), and then quality filtered and demultiplexed in QIIME (quantitative insights into microbial ecology) using default settings (Caporaso et al. 2010). Chimeric sequences were removed by UCHIME (Edgar et al. 2011). Operational Taxonomic Unit (OTUs) were picked using de novo OTU picking protocol with a 97% similarity threshold and singletons and doubletons were discarded in the QIIME software. Taxonomy assignment of OTUs was performed by comparing sequences to the Greengenes database (gg_13_5_otus). Alpha diversity analysis included Shannon index, Chao1, PD whole tree and observed species. Jackknifed beta diversity included both unweighted and weighted Unifrac distances calculated with 10 times of subsampling, and these distances were visualized by Principal Coordinate Analysis (PCoA) (Lozupone and Knight 2005). Mann–Whitney U test were used for significance test of alpha diversity. Permutational multivariate analysis of variance (PERMANOVA) was used for significance test of beta diversity difference between sample groups. Linear discriminant analysis coupled with effect size (LEfSe) was performed to identify the microbial taxa differentially represented between groups at genus level, which was a robust tool that focused not only on statistical significance but also biological relevance (Segata et al. 2011). The original sequencing data of raw reads were deposited in the sequence read archive of the National Center for Biotechnology (accession nos. PRJNA437450, SRP134182).

### Group specific quantitative real-time PCR

Fecal Microbial population was analyzed using group-specific primers (Bartosch et al. 2004; Franks et al. 1998; Koike and Kobayashi 2001; Lee et al. 1996; Matsuki et al. 2002, 2004; Rinttila et al. 2004), as listed in Additional file 1: Table S1. qPCR was performed on a Bio-Rad CFX96™ real-time PCR Detection System with CFX Manager Software version 2.0 (Bio-Rad Laboratories, USA). Each reaction was run in duplicate in a volume of 25 μL in low 96-well white PCR plates sealed with optical flat 8-cap strips (Bio-Rad Laboratories, USA). The reaction mixture consisted of 12.5 μL SYBR^®^ Premix Ex Taq™ II (TaKaRa Biotechnology, China), 1 μL of each primer (Invitrogen Life Technologies, China), and 1 μL of template DNA of fecal samples (non-template control used water instead). Amplification program included an initial denaturation at 95 °C for 5 min followed by 40 cycles of denaturation at 95 °C for 15 s, primer annealing at the optimal temperatures (Additional file 1: Table S1) for 30 s and primer extension at 72 °C for 30 s, 1 cycle of 95 °C for 1 min, 1 cycle at 55 °C for 1 min, and a stepwise increase of the temperature from 55 to 95 °C (at 10 s/0.5 °C) to obtain melt curve data. Data were collected at the extension step. Melting curves were checked after amplification and size of PCR products were verified by agarose gels in order to ensure correct amplification results. Standard curves were generated as described by Rinttila et al. (2004) and Schwab and Ganzle (2011).

## Results

### Sequencing metadata

After removing the low quality reads and chimeras, a total of 60,411 chimera-free high quality 16S rRNA gene sequences (V4–533–786 bp) were obtained, with an average of 5034.25 ± 178.35 sequences per sample, ranging from 4038 to 5321. These sequences, with average length of 252 bp, were assigned to a total of 28,467 operational taxonomic units (OTUs) based on 97% similarity. Each sample has 1404.25 ± 308.21 OTUs on average.

### Comparison of microbial community diversity and structures between wild and captive takins

**Fig. 1:**
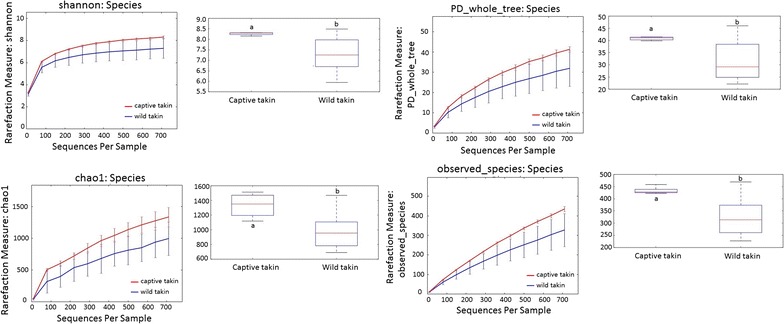
Differences in microbial community diversity and richness between wild and captive takins. Diversity and richness were measured by Shannon index, Chao1, PD whole tree and observed species respectively. Different lowercase letters above the boxplots indicate significant differences between groups (*P* < 0.05, Mann–Whitney U test)

**Fig. 2:**
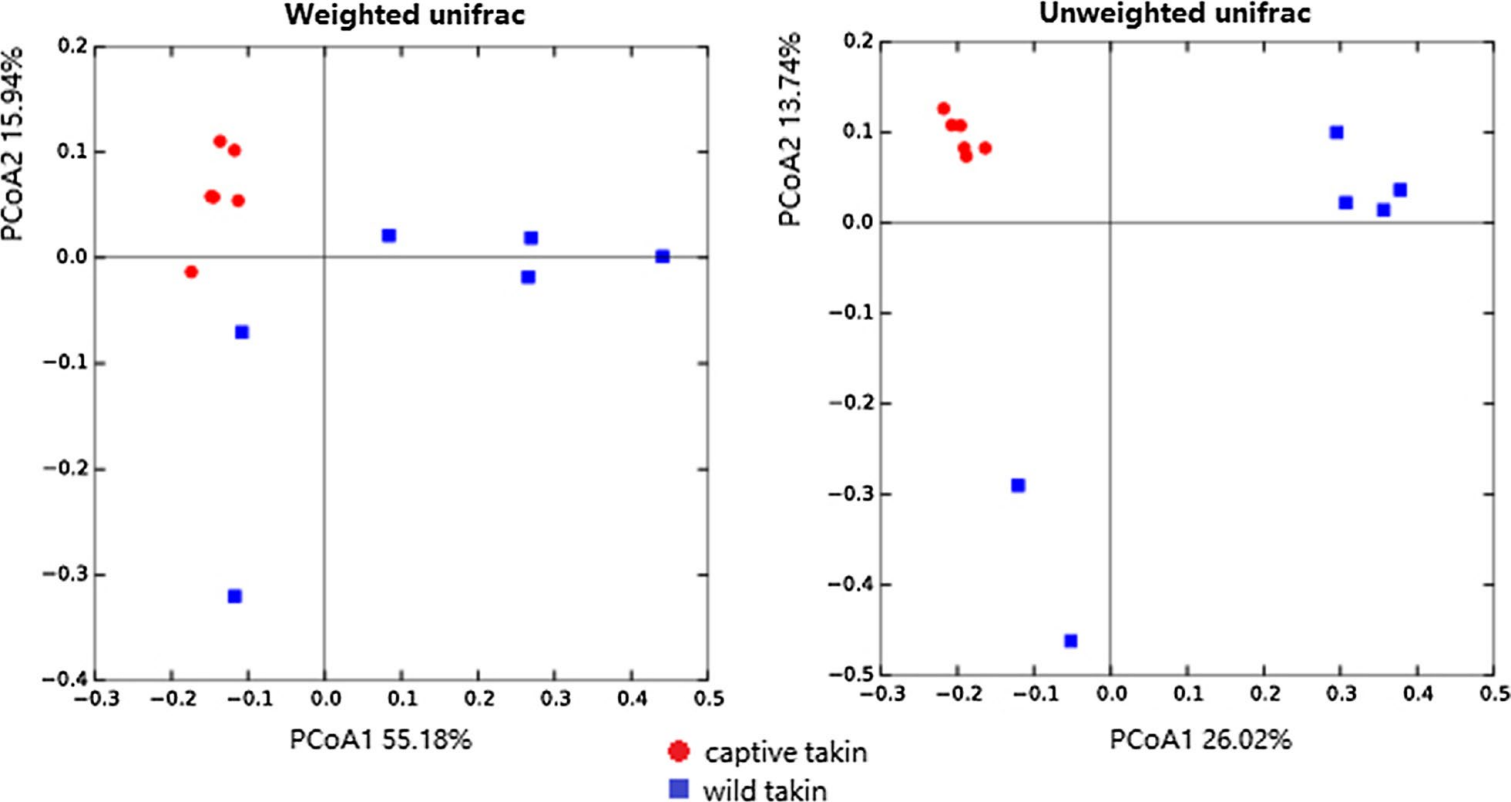
The PCoA analysis of microbial community in wild and captive takins based on weighted and unweighted Unifrac distance

Microbial community diversity was measured by Shannon index, Chao1, PD whole tree and observed species. All indices were significantly higher in the captive than in the wild takins (Fig. 1, Mann–Whitney U test, *P* < 0.05), which indicated a higher community diversity (both richness and evenness) of the intestinal microbiota in captive takins. Community structure of the intestinal microbiota also displayed significant separation between wild and captive takins. Both weighted and unweighted UniFrac distances were calculated and Principal Coordinate Analysis (PCoA) was applied to visualize the dissimilarities in community membership (Fig. 2 and Additional file 2: Fig. S1). Based on the membership, microbial communities from captive takins clustered together and separated from those from wild takins along principal coordinate axis. Notably, microbial communities of captive takins clustered tightly, while communities of wild takins were more dispersed. Permutational multivariate analysis of variance (PERMANOVA, F = 2.72, *P* = 0.005) revealed the differences in community membership between wild and captive takins were statistically significant.

**Fig. 3:**
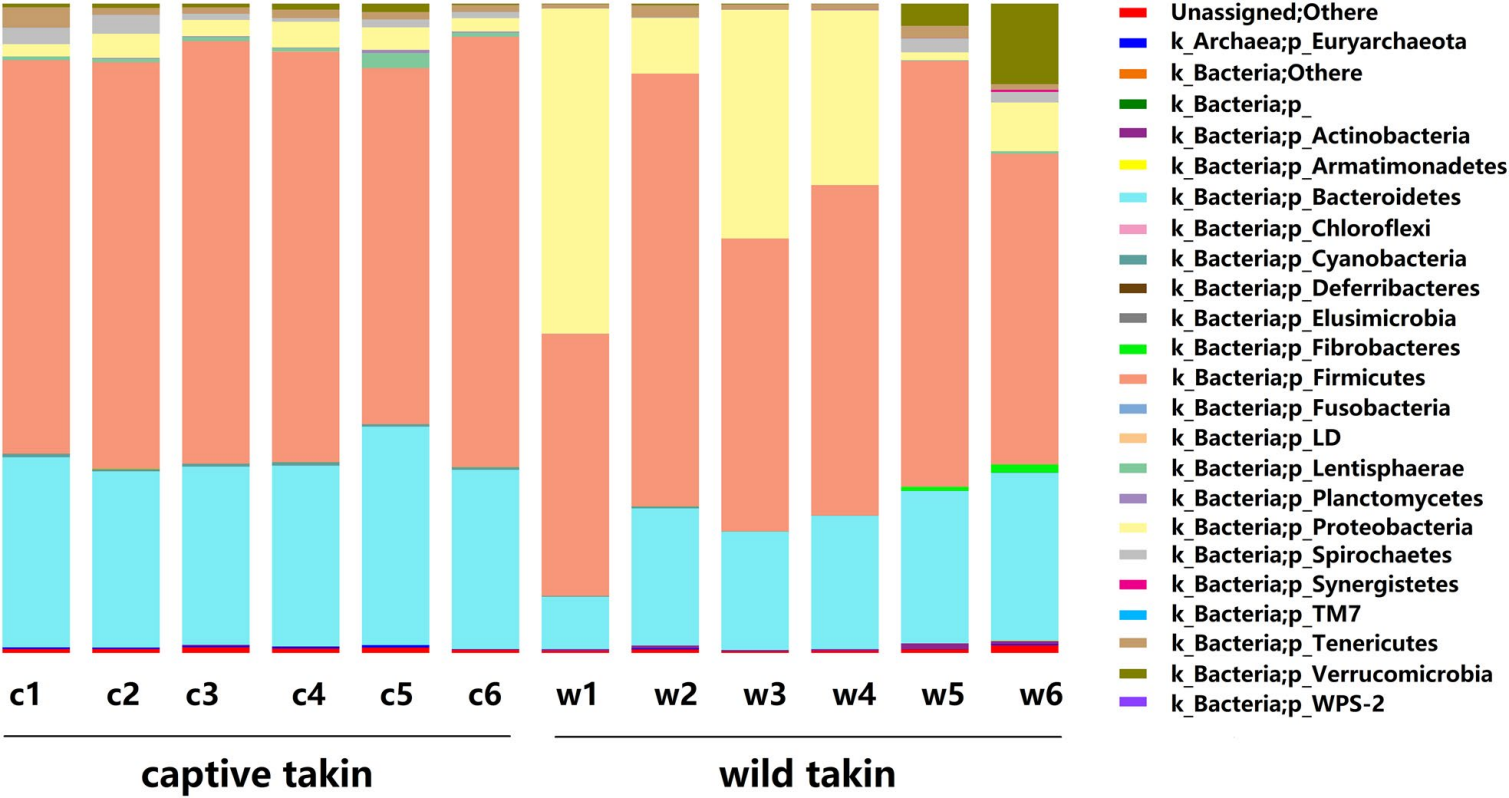
Relative abundance of microbial taxon at phylum level in the fecal microbiota from wild and captive takins

Wild takins possessed similar community structure to captive takins. The microbial communities of takins were mostly dominated by representatives from three phyla: *Firmicutes* (57.4%), *Bacteroidetes* (24.2%) and *Proteobacteria* (12.3%). At family/genus level, *Ruminococcaceae*, *Bacteroidaceae*, *Acinetobacter*, *Clostridium*, *Lachnospiraceae*, *Rikenellaceae*, *Bacillus*, *Comamonas* and *Spirochaetaceae* were dominant. However, the relative abundance of major phyla differed considerably between wild and captive takins. As shown in Fig. 3, the phylum *Bacteroidetes* contributed significantly more to the intestinal microbiota of captive takins (28.81 ± 2.4%) than wild takins (19.53 ± 6.2%) (Student’s *t* test, *P* = 0.007). The phylum *Firmicutes* also contributed more to the intestinal microbiota of captive takins (62.12 ± 4.1%) than wild takins (52.75 ± 10.9%), although these trends were not significant (Student’s t-test, P = 0.077). The phylum *Proteobacteria* contributed significantly more to the intestinal microbiota of wild takins (21.57 ± 19.0%) than captive takins (2.9 ± 0.9%) (Student’s t-test, *P* = 0.037).

**Fig. 4:**
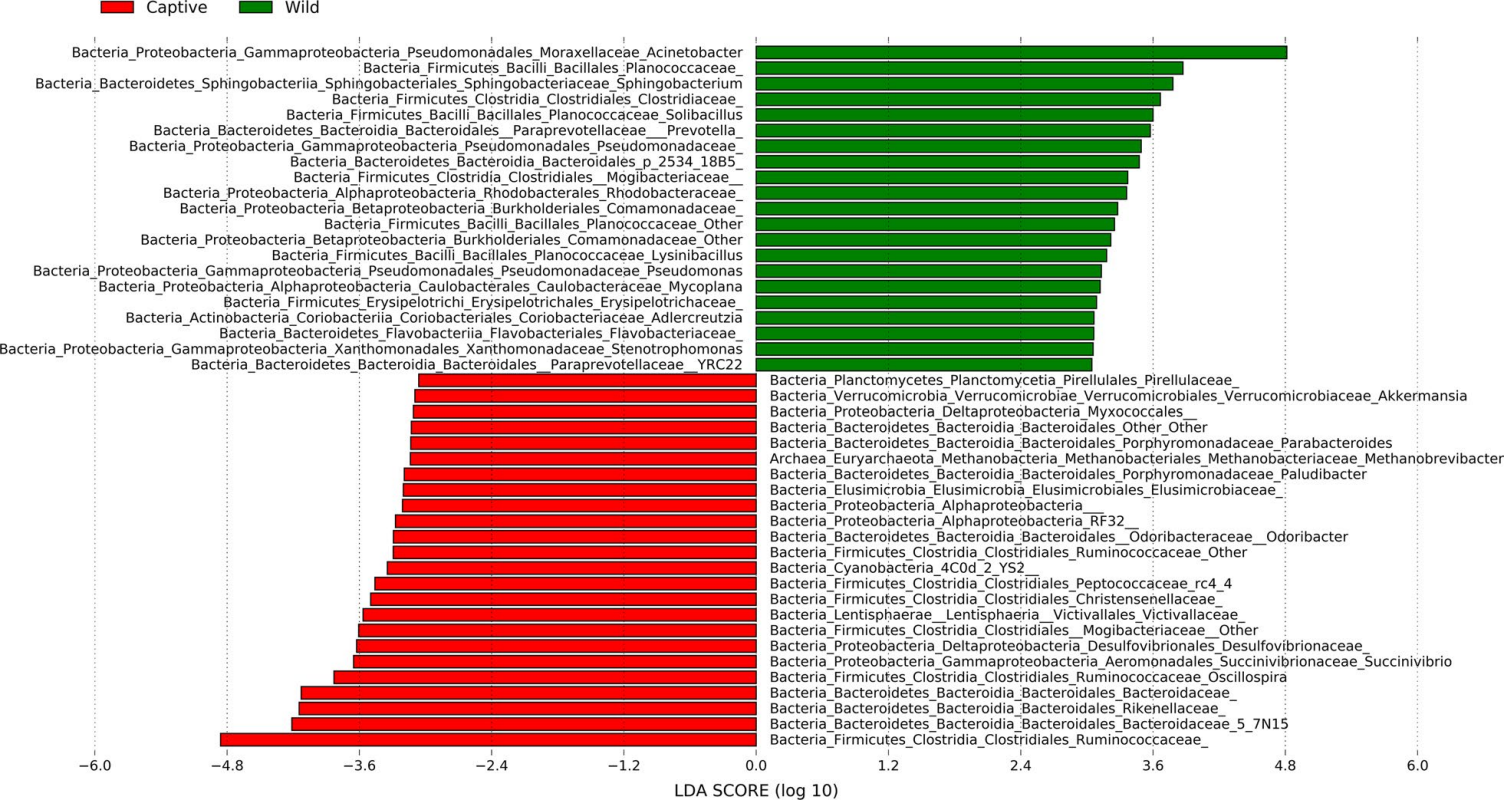
Genus differentially represented between wild and captive takins identified by linear discriminant analysis coupled with effect size (LEfSe)

The linear discriminant analysis effect size (LEfSe) was employed to identify specific genus that were differentially distributed between wild and captive takins. The results of LEfSe were shown in Fig. 4. A total of 45 genus were differentially represented between the two groups, with 21 more abundant in wild takins (e.g. *Acinetobacter*, *Sphingobacterium*, *Solibacillus*, *Lysinibacillus*, *Pseudomonas*, *Mycopiana*, *Adlercreutzia*, *Stenotrophomonas*, *YRC 22*) and 24 more abundant in captive takins (e.g. *Pirellulaceae*, *Akkermansia*, *Parabacteroides*, *Methanobrevibacter*, *Paludibacter*, *Odoribacter, Victivallaceae, Succinivibrio, Oscillospira*).

### The influence of individual factor on the intestinal microbiota

Since Illumina Miseq sequencing of partial 16S RNA genes could only provide the relative abundance of microbial taxa, fifteen pairs of group-specific primers were applied to quantify microbial populations in feces of takins. In order to explore the influence of individual factors (age, gender and sampling time) on the intestinal microbiota of takins, predominant microbial populations of three captive takins were monitored for 6 months.

**Fig. 5:**
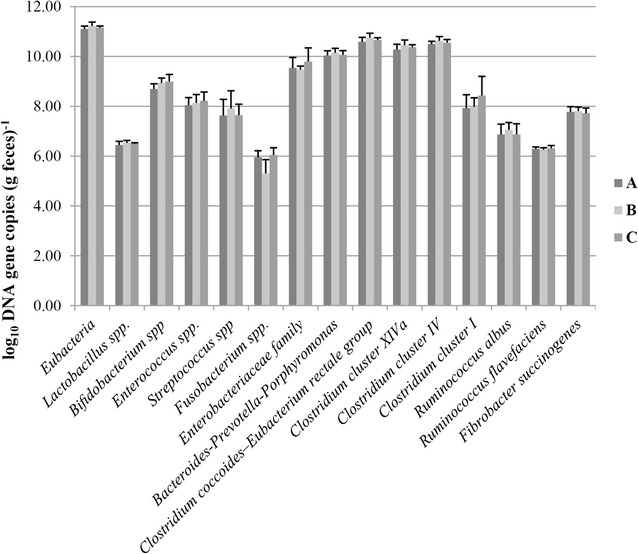
Average microbial populations in the feces of three takins over all sampling points. A, female takin; B, male takin; C, young male takin

As the quantification results show in Fig. 5, the number of total bacterial gene copies (Eubacteria) in takin feces ranged from 10.98 to 11.35 log_10_ DNA gene copies per gram feces. *Blautia coccoides*–*Eubacterium rectale* group represented the majority of the fecal bacterial population, with the average number of 10.67 ± 0.16 log_10_ DNA gene copies per gram feces. The *Bacteroides*–*Prevotella*–*Porphyromonas* group was also dominant in the feces of takins, which was present at 10.08 ± 0.17 log_10_ DNA gene copies per gram feces. The number of clostridia in feces of takins was also high. Among the clostridia, the *Clostridium* clusters IV and XIVa were generally present in equal numbers of approximately 10.09–10.75 log_10_ DNA gene copies per gram feces, even *Clostridium* cluster I was detected between 7.36 and 9.31 log_10_ DNA gene copies per gram feces. Three presently well-recognized major cellulolytic bacterial species were also detected, *Fibrobacter succinogenes* was most dominant (7.77 ± 0.18 log_10_ DNA gene copies per gram feces) among the three species, followed by *Ruminococcus albus* (6.93 ± 0.36 log_10_ DNA gene copies per gram feces) and *Ruminococcus flavefaciens* (6.29 ± 0.09 log_10_ DNA gene copies per gram feces). Besides, *Enterobacteriaceae* family was the predominant facultative anaerobe in the feces of takins with a population of 9.6 ± 0.4 log_10_ DNA gene copies per gram feces. *Bifidobacterium* spp., *Enterococcus* spp., *Streptococcus* spp., *Lactobacillus* spp. and *Fusobacterium* spp. also prevailed in the feces of takins (8.88 ± 0.25, 8.31 ± 0.13, 7.73 ± 0.51, 6.49 ± 0.1 and 5.77 ± 0.49 log_10_ DNA gene copies per gram feces, respectively). These results were generally consistent with the results of Illumina Miseq sequencing.

**Fig. 6:**
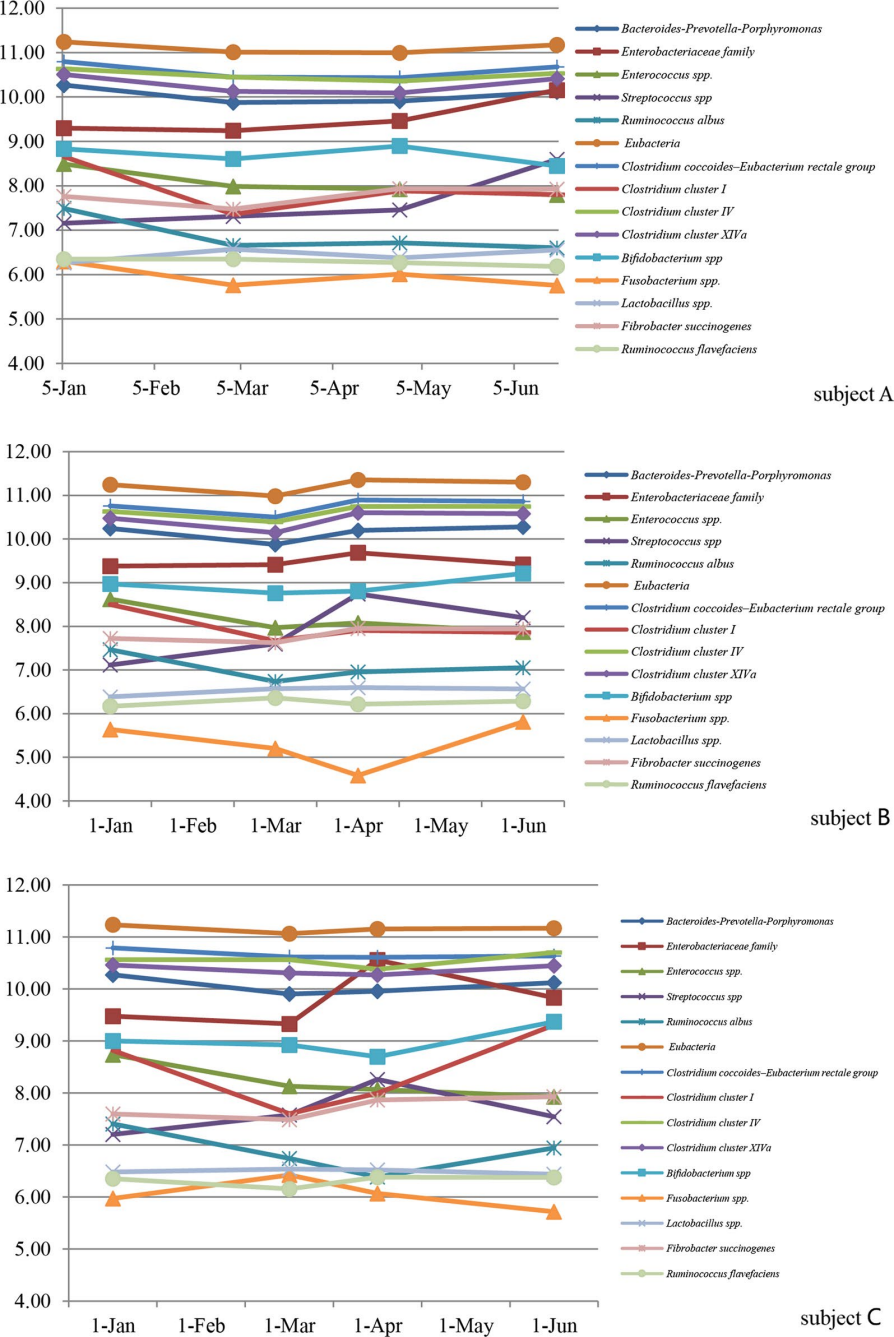
Microbial populations of three takins over a period of half year

From the perspective of the individual, the three takins (subject A, B and C) harbored similar microbial populations. No significant differences were observed in the average numbers of each group/species among the three individuals (Student’s t-test, *P* > 0.05). Differences of each group/species between individuals were all less than 1 log_10_ DNA gene copies per gram feces. Generally, volatility of microbial populations in the three takins were relatively small during our study period. Significant fluctuations only occurred in the numbers of some microbial taxa like *Streptococcus* spp., *Clostridium* cluster I, *Fusobacterium* spp., *Enterobacteriaceae* family and *Ruminococcus albus*, which all fluctuated more than 1 log_10_ DNA gene copies per gram feces during 6-month period (Fig. 6).

## Discussion

To our knowledge, the current work is the first comprehensive study to evaluate the fecal microbiota of the Sichuan takin. In order to effectively study their complex microbial community, Illumina Miseq sequencing and group-specific qPCR had been employed in this study. Based on the results of Illumina Miseq sequencing, the microbial communities of takins were mostly dominated by bacteria belonging to *Firmicutes*, *Bacteroidetes* and *Proteobacteria*, which comprised more than 90% of the total bacteria. These phyla were also dominant in feces from the cattle, muskoxen and sheep hindgut (De Oliveira et al. 2013; Salgadoflores et al. 2016; Zeng et al. 2017). At family/genus level, *Ruminococcaceae*, *Bacteroidaceae*, *Acinetobacter*, *Clostridium*, *Lachnospiraceae*, *Rikenellaceae*, *Bacillus*, *Comamonas*, *Oscillospira* and *Spirochaetaceae* constituted the major family/genus. Furet et al. also used qPCR for enumeration of fecal bacteria in human and farm animal (rabbits, goats, horses, pigs, sheep and cows) (Furet et al. 2009). In our study, the same range of populations for the total bacteria and dominant/subdominant bacterial groups like *Clostridium*, *Blautia coccoides* and *Bacteroides*–*Prevotella group* in sheep and cows were also found in takins. Thus, the fecal microbiota of the takin quite resembled that of cattle, muskoxen and sheep. From the phylogenetic perspective, the takin is in an intermediate position between the cattle and sheep. The takin has similar body size and metabolic requirements to cattle, while their external characteristics are rather similar to sheep. In captivity, the takin even had similar diet and living environment to these animals. All these factors might contribute to captive takin possessing a similar microbial profiles (Demment and Van Soest 1985; Ley et al. 2008b).

Like other herbivores, most dominant bacteria of takins were fiber-digesting bacteria. *Ruminococcaceae* is one of the most predominant family in the feces of takins. Some *Ruminococcaceae* played a key role in cellulose degradation (David et al. 2015; Rincon et al. 2001). As members of this family, *Ruminococcus flavefaciens*, *Ruminococcus albus* were presently recognized as the major cellulolytic bacterial species found in the rumen (Rincon et al. 2005; Wanapat and Cherdthong 2009). *Oscillospira* was prevalent in the rumen and hindgut of several herbivores and it is involved in anaerobic fermentation as well (Mackie et al. 2003). *Clostridium* was another common and diverse genus in takins. In *Clostridium*, *Clostridium* cluster XIVa (*Blautia coccoides* group) and cluster IV (*Clostridium leptum* subgroup) were generally regarded responsible for the degradation of complex carbohydrates (Burrell et al. 2004). Previous study has also found that putative genes coding two cellulose-digesting enzymes and one hemicellulose-digesting enzyme were recovered in *Clostridium* group I from intestinal microbes of the panda, which was thought to be an evidence of cellulose metabolism by the giant panda intestinal microbiome (Zhu et al. 2011). The genus *Eubacterium* was the important constituent of *Blautia coccoides*–*Eubacterium rectale* group. Some members affiliated with *Eubacterium* in the rumen were reported to play the major role in mediating the butyrogenic effect of fermentable dietary carbohydrates (Mitsumori and Minato 1995). In addition, members of the *Bacteroides* genus were generally considered as the most important group in terms of pectin and lignin degradation, due to their high numbers and nutritional versatility (Akin 1988; Kuritza et al. 1986; Salyers et al. 1977; Weaver et al. 1992). The genus *Prevotella* is previously classified as member of the genus *Bacteroides*. These species possess oligosaccharolytic and xylanolytic activities to degrade individual sugars, and also utilize amino acids, and small peptides for their growth, which play a vital role in ruminal carbohydrate and protein fermentation (Marteau et al. 2001). Such as large population of fiber-digesting bacteria would enable the takin a powerful ability to utilize fibrous plant materials as nutrients, as those naturally consumed by wild members. Overall, the microbial community of takins were compatible with their diets structures and metabolic capacities.

To investigate the influence of diets and environmental exposures on the intestinal microbiota, we compared fecal microbiota in takins from Labahe Nature Reserve and Chengdu Zoo. Distinctive microbiotas between wild and captive takins were observed based on community diversity and membership. Compared with wild takins, captive takins had significantly higher community diversity (both richness and evenness). Interestingly, the variation of microbial community structures in wild takins was significantly higher than that in captive takins. Diet has been considered as a major driver shaping fecal microbial community composition (Ley et al. 2008a). In captivity, takins are fed a constant and balanced diet, while free-ranging wild takin typically graze on various plants. It could be cautiously speculated that dietary items drove the divergence in the microbiota between wild and captive takins. Meanwhile, random and heterogeneity of diets in wild takins contributed to their large dispersion in microbial community structures. Comparatively, individual factors exerted less influence on microbial community than captivity. As revealed by group-specific qPCR, microbial communities of the three captive takins were relatively similar to each other and stable during our study period. In the few comparable longitudinal studies, microbial communities in the fecal samples of other herbivores (panda, cows and calves) were also stable (Ibekwe et al. 2003; Wei et al. 2007). In contrast, microbial communities present in polar bear feces varied to a larger extent (Schwab and Ganzle 2011). Polar bears harbour simple intestinal physiology and fast digestion times. Therefore, we speculated that relative stability of microbial communities in takins might be caused by their herbivorous diet structure and complex but stable intestinal physiology. Especially for captive takins, they had the same diet composition and the close genetic relationship among individuals. Nevertheless, we still observed an appreciable effect of sampling time on stability of microbial communities in captive takins, which might be caused by seasonal variation in nutritive component of forage grasses and the potential impact of climate on animal physiology.

In summary, we presented the first characterization of the intestinal microbiota in the takins Illumina Miseq sequencing. Distinctive microbiotas between wild and captive takins were observed as a result of their different diet and living environment. Further group-specific qPCR showed that microbial communities of the three takins were relatively similar to each other and stable during our study period, while sampling time had an appreciable effect on microbial communities of the takin. All these results will allow for a better understanding of the putative role of the takin intestinal bacteria in the health and physiological function of the host animal. Of course, the ability to maintain and successfully breed animals threatened with extinction in zoos or protected nature reserves still requires more intimate knowledge of their intestinal microbial ecology and the interrelationships between their native bacteria, diet and nutrient harvest.

## Additional files

**Additional file 1: Table S1.** Primers used for group-specific quantitative real-time PCR.

**Additional file 2: Fig. S1.** The PCoA analysis of microbial community in wild and captive takins based on weighted and unweighted Unifrac distance (PC1vsPC3 and PC2vsPC3).

## Abbreviations

qPCR: quantitative real-time PCR
CFU: colony-forming unit
PCoA: Principal Coordinate Analysis
PERMANOVA: permutational multivariate analysis of variance
LEfSe: linear discriminant analysis coupled with effect size
