# Supplementary material for: Microbial community and diversity in the feces of Sichuan takin (Budorcas taxicolor tibetana) as revealed by Illumina Miseq sequencing and quantitative real-time PCR
Source: AMB Express. 2018 Apr 27;8:68. doi: 10.1186/s13568-018-0599-y (PMC5924506; doi:10.1186/s13568-018-0599-y)
Supplement: Supplementary file 1 — Additional file 1: Table S1. Primers used for group-specific quantitative real-time PCR. [file 13568_2018_599_MOESM1_ESM.docx]

Table S1. Primers used for group-specific quantitative real-time PCR.

| Targeted bacterial group (primer size/bp) Orientation Primer sequence (5’-3’) Annealing temp (°C) Reference | | | | |
| --- | --- | --- | --- | --- |
| Total bacteria/Eubacteria (200) | F | CGGYCCAGACTCCTACGGG | 60 | (Lee et al., 1996) |
|  | R | TTACCGCGGCTGCTGGCAC |  |  |
| *Lactobacillus* spp. (341) | F | AGCAGTAGGGAATCTTCCA | 55 | (Rinttila et al., 2004) |
|  | R | CACCGCTACACATGGAG |  |  |
| *Enterococcus* spp. (144) | F | CCCTTATTGTTAGTTGCCATCATT | 52 | (Rinttila et al., 2004) |
|  | R | ACTCGTTGTACTTCCCATTGT |  |  |
| *Biﬁdobacterium* spp. (243) | F | TCGCGTCYGGTGTGAAAG | 62 | (Rinttila et al., 2004) |
|  | R | CCACATCCAGCRTCCAC |  |  |
| *Streptococcus* spp. (485) | F | AGAGTTTGATCCTGGCTCAG | 52 | (Franks et al., 1998) |
|  | R | GTTAGCCGTCCCTTTCTGG |  |  |
| *Clostridium* cluster XIVa (438-441) | F | AAATGACGGTACCTGACTAA | 60 | (Matsuki et al., 2002) |
|  | R | CTTTGAGTTTCATTCTTGCGAA |  |  |
| *Clostridium* cluster IV (130) | F | GCACAAGCAGTGGAGT | 60 | (Matsuki et al., 2004) |
|  | R | CTTCCTCCGTTTTGTCAA |  |  |
| *Clostridium* cluster I(120) | F | ATGCAAGTCGAGCGAKG | 60 | (Rinttila et al., 2004) |
|  | R | TATGCGGTATTAATCTYCCTTT |  |  |
| *Bacteroides-Prevotella-Porphyromonas* (140) | F | GGTGTCGGCTTAAGTGCCAT | 52 | (Rinttila et al., 2004) |
|  | R | CGGAYGTAAGGGCCGTGC |  |  |
| *Enterobacteriaceae* family (195) | F | CATTGACGTTACCCGCAGAAGAAGC | 52 | (Bartosch et al., 2004) |
|  | R | CTCTACGAGACTCAAGCTTGC |  |  |
| *Blautia coccoides-Eubacterium rectale* group (429) | F  R | CGGTACCTGACTAAGAAGC  AGTTTYATTCTTGCGAACG | 60 | (Rinttila et al., 2004) |
| *Ruminococcus albus* (175) | F | CCCTAAAAGCAGTCTTAGTTCG | 52 | (Koike and Kobayashi, 2001) |
|  | R | CCTCCTTGCGGTTAGAACA |  |  |
| *Fibrobacter succinogenes* (445) | F | GGTATGGGATGAGCTTGC | 56 | (Koike and Kobayashi, 2001) |
|  | R | GCCTGCCCCTGAACTATC |  |  |
| *Ruminococcus flavefaciens* (835) | F | GGACGATAATGACGGTACTT | 59 | (Koike and Kobayashi, 2001) |
|  | R | GCAATCYGAACTGGGACAAT |  |  |
| *Fusobacterium* spp.(273) | F | CWAACGCGATAAGTAATC | 55 | (Rinttila et al., 2004) |
|  | R | TGGTAACATACGAWAGGG |  |  |
|  | | | | |

Bartosch S, Fite A, Macfarlane GT, Mcmurdo MET (2004) Characterization of bacterial communities in feces from healthy elderly volunteers and hospitalized elderly patients by using real-time PCR and effects of antibiotic treatment on the fecal microbiota. Appl Environ Microb 70:3575-3581. doi 10.1128/AEM.70.6.3575-3581.2004.

Franks AH, Harmsen HJM, Raangs GC, Jansen GJ, Schut F, Welling GW (1998) Variations of Bacterial Populations in Human Feces Measured by Fluorescent In Situ Hybridization with Group-Specific 16S rRNA-Targeted Oligonucleotide Probes. Appl Environ Microb 64:3336-3345. doi: 10.12938/bifidus1996.19.79.

Koike S, Kobayashi Y (2001) Development and use of competitive PCR assays for the rumen cellulolytic bacteria: *Fibrobacter succinogenes*, *Ruminococcus albus* and *Ruminococcus flavefaciens*. Fems Microbiol Lett 204:361-366. doi: 10.1111/j.1574-6968.2001.tb10911.x.

Lee DH, Zo YG, Kim SJ (1996) Nonradioactive method to study genetic profiles of natural bacterial communities by PCR-single-strand-conformation polymorphism. Appl Environ Microb 62:3112-3120.

Matsuki T, Watanabe K, Fujimoto J, Miyamoto Y, Takada T, Matsumoto K, Oyaizu H, Tanaka R (2002) Development of 16S rRNA-Gene-Targeted Group-Specific Primers for the Detection and Identification of Predominant Bacteria in Human Feces. Appl Environ Microb 68:5445-5451. doi: 10.1128/AEM.68.11.5445-5451.2002.

Matsuki, T., Watanabe, K., Fujimoto, J., Takada, T., and Tanaka, R (2004) Use of 16S rRNA Gene-Targeted Group-Specific Primers for Real-Time PCR Analysis of Predominant Bacteria in Human Feces. Appl Environ Microb 70: 7220-7228. doi:10.1128/AEM.70.12.7220-7228.2004.

Rinttila T, Kassinen A, Malinen E, Krogius L, Palva A (2004) Development of an extensive set of 16S rDNA-targeted primers for quantification of pathogenic and indigenous bacteria in faecal samples by real-time PCR. J Appl Microbiol 97:1166-1177. doi: 10.1111/j.1365-2672.2004.02409.x.
