# Supplementary figures and images for: Microbial community and diversity in the feces of Sichuan takin (Budorcas taxicolor tibetana) as revealed by Illumina Miseq sequencing and quantitative real-time PCR
Source: AMB Express. 2018 Apr 27;8:68. doi: 10.1186/s13568-018-0599-y (PMC5924506; doi:10.1186/s13568-018-0599-y)

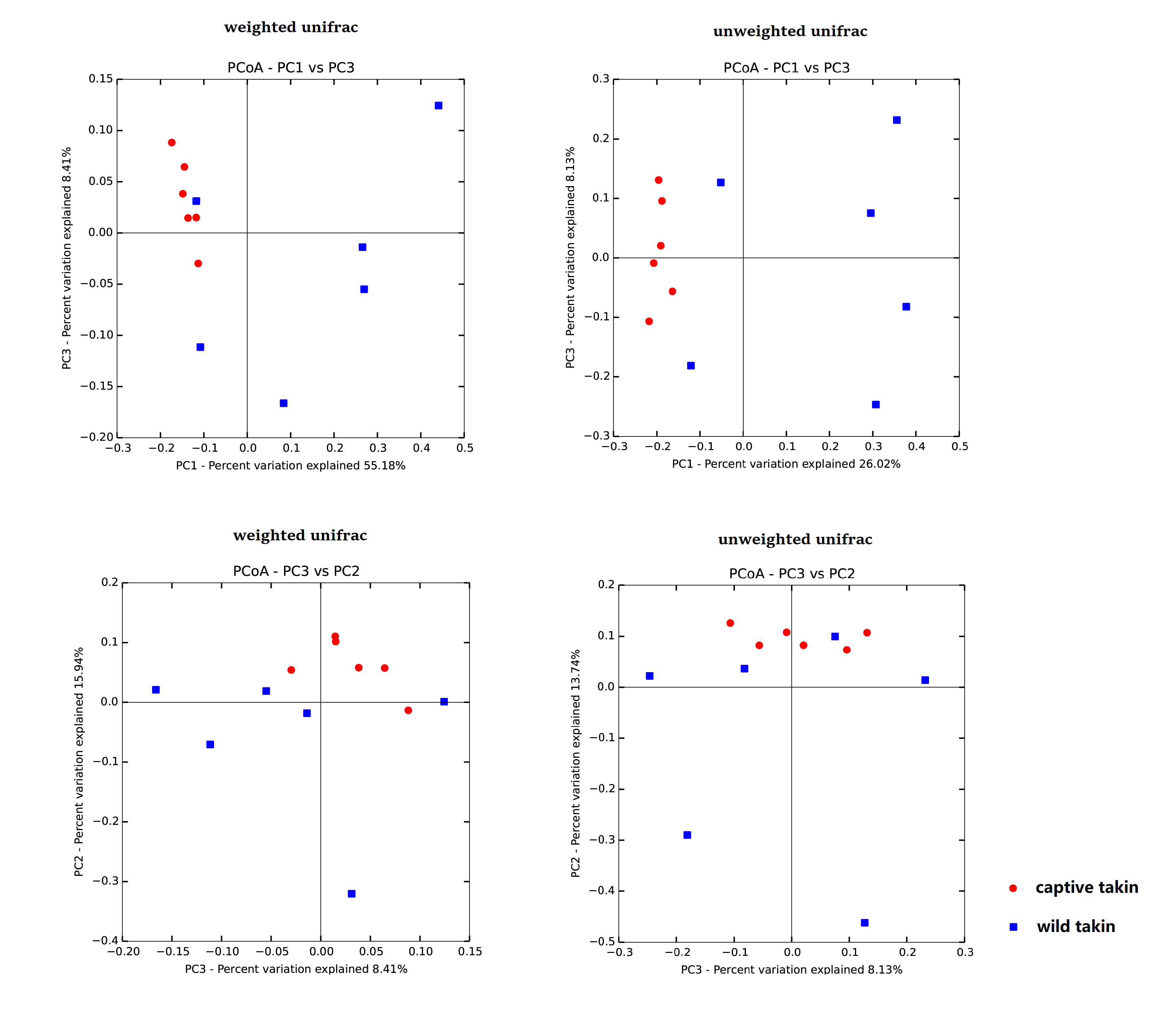

Supplement: Supplementary file 2 — Additional file 2: Fig. S1. The PCoA analysis of microbial community in wild and captive takins based on weighted and unweighted Unifrac distance (PC1vsPC3 and PC2vsPC3). [file 13568_2018_599_MOESM2_ESM.png]
